# Bendable Substrates of Cellulose Nanocrystals for Triboelectric Nanogenerators

**DOI:** 10.1021/acsanm.5c01087

**Published:** 2025-05-03

**Authors:** Amit Kumar Sonker, Charchit Kumar, Hannah Tideland, Satyaranjan Bairagi, Nirmal Kumar Katiyar, Daniel M. Mulvihill, Gunnar Westman

**Affiliations:** † BA5409, Cellulose Films and Coatings, BA54 Biomaterials Processing and Products, VTT Technical Research Centre of Finland, Tietotie 4E, Espoo 02150, Finland; ‡ Department of Chemistry and Chemical Engineering, Chalmers University of Technology, Gothenberg 41296, Sweden; § Department of Chemistry and Molecular Biology, University of Gothenberg, Gothenberg 40530, Sweden; ∥ Department of Chemistry, Amity Institute of Applied Science, Amity University, Noida 201313, Uttar Pradesh, India; ⊥ Wallenberg Wood Science Centre, Chalmers University of Technology, Gothenberg 41296, Sweden; # Materials and Manufacturing Research Group, James Watt School of Engineering, University of Glasgow, Glasgow G12 Q88, U.K.; ¶ Biomedical and Mobile Health Technology Lab, Department of Health Science and Technology, ETH Zurich, Lengghalde 5, Zurich 80008, Switzerland

**Keywords:** cellulose nanocrystals, plasticizer, counterion, crystallinity, triboelectric nanogenerators

## Abstract

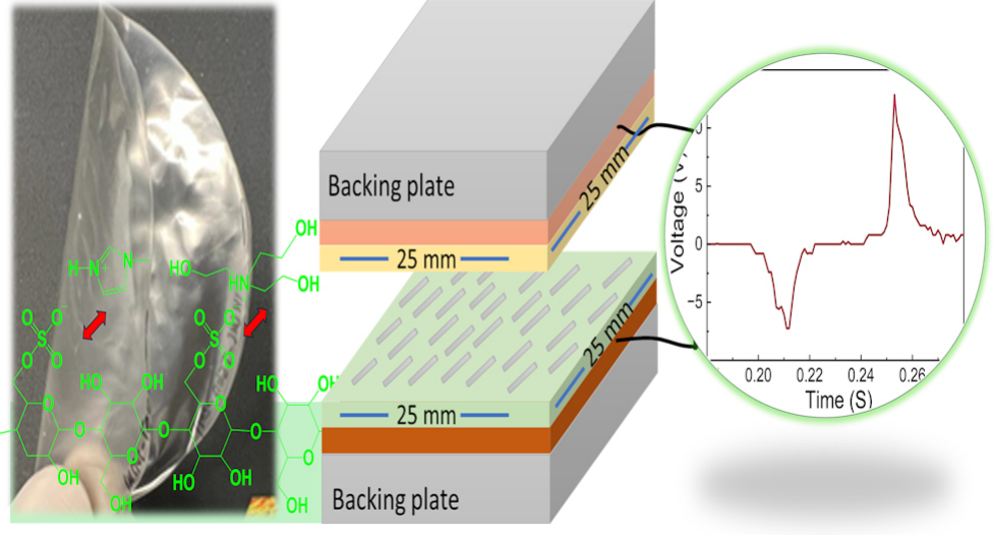

Triboelectric nanogenerators (TENGs) effectively convert mechanical energy to electric power or signals. In the presented work, films of sulfated cellulose nanocrystals (CNC) in combination with additives, triethanolamine (TEOA), and methylimidazole (MI) were turned into a tribo-positive layer in combination with polyethylene terephthalate (PET) (tribo-negative layer) to develop CNC-PET TENG. The additives improved the bendability of the formed films. Under optimized additive concentrations, cellulose nanocrystals (CNC) film with 4 wt % TEOA and 4.5 wt % MI shows tensile strength of 68 MPa with 4.8% elongation at break values in comparison to 47 MPa strength and 1% elongation at break values for neat CNC films. The CNC-PET film-based vertical contact-separation TENG device shows output voltages of 80 and 123 V at frequencies of 4 and 8 Hz, respectively. The TENG based on modified CNC films was stable for 4000 contact-separation cycles. Unlike TENG based on CNF and other nanocellulose composites, in this work, CNC films consist of more than 90 wt % cellulose and show good electromechanical performance, which makes them promising candidates as substrates for flexible electronics with recyclability over earlier published reports on cellulose composite with synthetic additives described in the introduction section.

## 1 Introduction

Plastics such as polyethylene terephthalate (PET) and polyimide (PI), along with ultrathin glass and metal foil, currently dominate the market for substrates used in flexible electronics.^1^ However, as the demand for more sustainable materials grows, replacing existing plastic materials and developing biobased materials with new functionalities is necessary. Cellulose-based materials offer several advantages, including biodegradability, renewability, and nontoxicity. These materials have been combined with functional inks to create conductive substrates for various applications, such as photovoltaics, sensors, batteries, and OLEDs.^2,3^ Cellulose fibers in paper have been used as a flexible substrate in electronics since the 1960s^4^ and remain the most commonly used cellulose-based substrate for printed circuits due to its availability, low cost, and higher heat tolerance compared to PET. However, paper has drawbacks such as high roughness, porosity, and particularly high water absorption.^2^ The development of nanocellulose, which consists of separated elementary fibrils of semicrystalline cellulose particles, such as cellulose nanocrystals (CNC) and cellulose nanofibrils (CNF), has made it possible to overcome these disadvantages. The nanocellulose particles are anisotropic yet exhibit uniformity in all directions. Due to their small size (approximately 5 nm width), they can form smooth films with low porosity. While CNF films are more flexible than CNC films, the latter are easier to form into films using coating and solution casting methods since CNC suspensions can be processed and cast at a concentration almost 10 wt % higher than CNF suspensions due to their lower viscosity. CNC films also have the advantage of absorbing less water compared with CNF films. Since CNC crystallites are inherently stiff and the mesophases form through closely packed CNCs resembling a brick-like structure, the contact between crystallites lacks flexibility, resulting in brittle films. Flexibility can be introduced by coassembling CNC with polymer precursors in nanocomposites^5−8^ or by adding small molecular plasticizers such as zwitterionic surfactants,^9^ glycerol (GLY),^5,10^ glucose,^5,6,10,11^ or citric acid.^12^ Moreover, in a recent publication by Tideland et al., it was shown that the bendability of CNC films could be improved by varying counterion and processing conditions. Specifically, it was found that applying presonication with sufficient energy is crucial as an initial step to reduce the inherent brittleness of CNC films produced by solution casting, Presonication also helps to decrease wettability (see Figure S7, Supporting Information).^13^ By incorporating bendability into the films, they become more ductile, enabling their application in systems subject to vibrations. These properties are particularly advantageous for sustainable energy solutions, as they allow for efficient energy harvesting in triboelectric nanogenerators (TENGs), which generate electricity from mechanical energy through the coupling of electrostatic induction and triboelectrification. A typical TENG device consists of two electrodes and at least one insulating material that serves as the triboelectric active layer. Studies have shown that the performance of TENGs is directly influenced by the charge density on the contact surface. Therefore, the active membrane should facilitate charge separation and charge transfer to the surface, which needs to be a smooth surface to maximize the contact area. One common method of surface smoothness is the addition of poly(dimethylsiloxane) (PDMS) to enhance energy output.^14−18^

Cellulose fibers inherently exhibit low triboelectric properties. As a result, developing cellulose-based triboelectric nanogenerators involves incorporating a substrate that enhances the electric effect.^19,20^ Nanocellulose, particularly nanocrystalline cellulose, exhibits a higher degree of alignment in its cellulose polymers compared to fibers. This increased alignment enhances the dipole moments generated by the orientation of the cellulose polymers, resulting in superior dielectric properties compared with cellulose fibers. Additionally, the crystallites are organized into ordered mesophases, further improving the dielectric performance. This enables the creation of nanocellulose tribolayers that are well-suited for TENG devices.^21,22^ Zhang et al. have reported the fabrication of a high-performance TENG device based on cellophane and regenerated cellulose film with an output of 300 W m^–2^.^23^ Yao et al. have shown how chemical functionalization of natural cellulose with nitro and methyl increases the triboelectric output, and when paired with the current output level, the results are similar to those obtained from fluorinated ethylene propylene.^19^ Another report from Yao et al. demonstrated the fabrication of triboelectric nanogenerators and power boards using cellulose nanofibrils.^24^ Fatma et al. used bacterial cellulose for a TENG made from polydopamine-BC, polypyrrole-BC and SiO_2_-BC tribolayers.^25^ Kim et al. reported an all-in-one TENG based on the silver nanowire/CNF composite.^26^ Also, there are numerous reports available on CNF (cellulose nanofibrils) and composites of CNF as tribolayers in TENG devices,^21,22^ but there are only a few reports on CNC-based TENG devices. Peng et al. have published a few articles which show the use of CNC in composite with PDMS, PVDF, and PU to improve the triboelectric performance of TENG.^27−29^

**Scheme 1:**
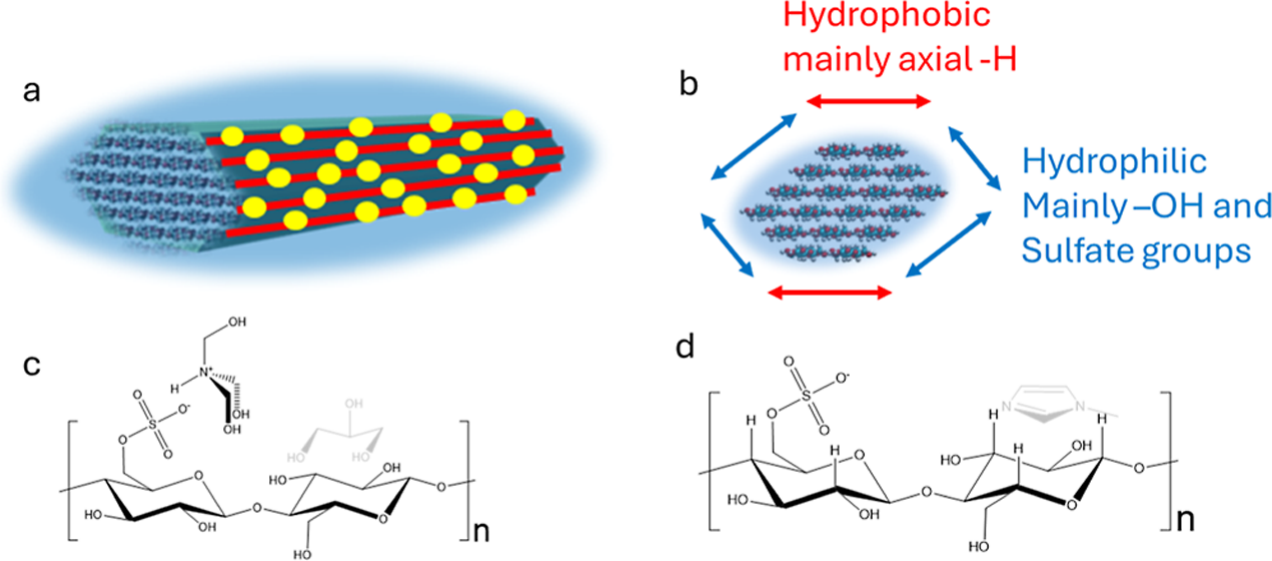
Schematic Presentation of (a) Nanocrystalline Cellulose (CNC) Yellow Spots is the Sulfate Groups on the Vertical Planes; (b) Cross-Section of CNC with Hydrophobic and Hydrophilic Planes; (c) Vertical Plane; GLY Coordination to Hydroxyl Groups and TEOA Coordination to Sulfate Groups; and (d) Imidazole Assumed to Coordinate to Axial –H Groups

The primary aim of this study was to develop bendable films from cellulose nanocrystal suspensions and investigate their potential as tribolayer materials in TENG devices. While there are few reports on pure CNC films, those that exist typically show much lower elongation at break values (≤2%).^30,31^ In this work, we aim to create films with higher elongation to improve their flexibility and mechanical durability, which are crucial for enhancing the performance and longevity of TENG devices under continuous mechanical stress. To find bendable and less fragile films with higher elongation, the sulfated CNC was mixed with three different additives: GLY, triethanolamine (TEOA), and *N*-methyl imidazole (MI). These additives are selected to achieve minor separations of the crystallites but are expected to have different types of coordination to the surface of CNC, assuming that GLY would coordinate with the hydroxyl groups, TEOA with the sulfate groups, and MI with the hydrophobic top-bottom of CNC, similar to CH/π interactions reported for aromatics^32^ (Scheme 1). According to the proposed hexagonal cross-section of CNC, the hydroxyl and sulfate groups decorate the vertical, two (110) and two (1–10) planes, making them hydrophilic. On the other hand, the top and bottom planes, (200), present axial CH groups from the glucose units, making them hydrophobic.^33,34^ Overall, the addition of GLY, TEOA, and MI to CNC films acts as plasticizers, enhancing the flexibility and uniformity of the films.

## 2 Materials and Methods

### 2.1 Materials

Microcrystalline cellulose (MCC) Avicel PH101, sulfuric acid (98%), TEOA, GLY, and MI were purchased from Sigma-Aldrich (now Merck), USA.

### 2.2 Methods

#### 2.2.1 Synthesis of Cellulose Nanocrystals

Cellulose Nano Crystals (CNC) were prepared by the established method of acid hydrolysis of MCC with 64 wt % sulfuric acid and the following purification method as described in our latest reports^13,35,36^ (see Supporting Information, Figure S9). The sulfate content on CNC was measured through potentiometric titration to be 275 μmol/g.

#### 2.2.2 Synthesis of Cellulose Nanocrystals, CNC, Films

30 mL of 2 wt % CNC suspensions in 50 mL of polypropylene beakers were vigorously stirred for 15 min by magnetic stirring (stirring break up lumps-aggregates); then, the suspensions were sonicated for 5 min using a probe sonicator at 40% amplitude (20 kHz, 500 W), resulting in a transparent suspension. The suspension was transferred into a polystyrene Petri dish and dried at 45 °C in a hot air oven for 1 day. After drying, films were peeled off, having a thickness of ∼80 μm, measured through a Mitutoyo micrometre.

#### 2.2.3 Synthesis of CNC Films with GLY and TEOA as Additives

To a CNC suspension of 2 wt %, 30 mL, 10, 20, or 25 wt % of plasticizers with respect to CNC was added [60, 120, or 150 mg of GLY or TEOA]. The mixture was vigorously stirred for 15 min by magnetic stirring, then sonicated for 5 min in the same as the CNC films in 2.2.2 was prepared. A screening study to find the correlation between the molar amount of TEOA and mechanical properties were done. The screening was performed on films prepared with varying amounts of TEOA relative to the sulfate groups. The sulfated CNC had a sulfate concentration of 275 μ mol/g; thus, 30 mL of 2 wt % (0.6 g CNC) has 165 μmol of sulfate groups. The molar concentrations of –OSO_3_H/TEOA were varied from 1:0.6 to 1:6 mol equivalents with a 0.6 mol equivalent interval, which is equivalent to 2.5 to 25 wt % concentrations with an interval of 2.5 wt %. All the suspensions were mixed as described in the preparation of CNC films, first mixed for 15 min by magnetic stirring and then sonicated for 5 min. The films were dried at 45 °C in a hot air oven for 1 day and conditioned at 50% RH. The measured thickness of these films varied from 90 to 100 μm measured through a Mitutoyo micrometer.

#### 2.2.4 CNC Films with TEOA and MI as Additives

Films containing both TEOA , assumed to coordinate to sulfate groups, and MI, assumed to coordinate to the more hydrophobic phases of CNC crystallites, were prepared as described for previous films. MI was used with concentration in the molar ratios (1:2), (1:4), and (1:6) with respect to the amount of TEOA molecules. Thus, the overall molar ratios were CNC–OSO_3_H/TEOA:MI as (1:1:2), (1:1:4), and (1:1:6). The measured thickness of these films varied from 110 to 120 μm measured through a Mitutoyo micrometre.

## 3 Characterization of CNC and CNC Films with Additives

### 3.1 Attenuated Total Reflectance-Fourier Transform Infrared (ATR-FTIR) Spectroscopy

The films were characterized by FTIR spectroscopy using a PerkinElmer Frontier instrument installed with an attenuated total reflection accessory. All cellulose powder and film samples were dried and scanned in the transmittance mode with a resolution of 2 cm^–1^ with 32 scans.

### 3.2 Mechanical Properties of CNC and Modified CNC Films

Instron UTM was used to measure the mechanical properties of CNC and CNC-additive films. ASTM D882-12 was considered for specimen preparation. The films were cut in the form of rectangular strips of dimensions 6 cm (length) × 5 mm (width) × ∼0.08 mm (thickness). A total of 5 specimens were tested for each sample with a strain rate of 0.5 mm/min. The samples were conditioned at 50% RH at 25 °C for 1 day prior to tensile testing.

### 3.3 Thermogravimetric Analysis

Thermogravimetric Analysis (TGA) was performed on the cellulose samples in the temperature range of 25–500 °C using a 3+ Star System from Mettler Toledo with a heating rate of 10 °C/min under nitrogen gas to measure the thermal stability of the samples. To highlight the difference in decomposition temperatures between the samples, the first derivative of the TGA plot was used as a differential thermal analysis (DTGA).

### 3.4 PET-CNC TENG Fabrication and Triboelectric Measurement

**Figure 1:**
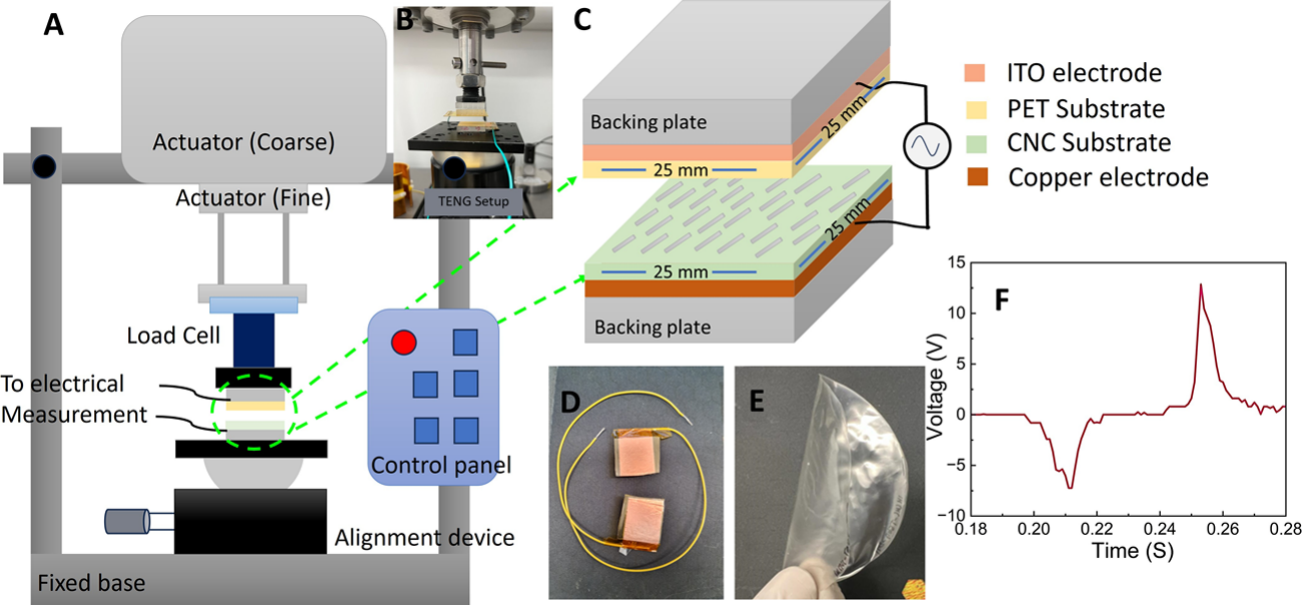
(A) Simplified schematic of custom-built Instron electro-pulse test rig, (B) real setup of the triboelectric nanogenerator (TENG), and (C) simplified schematic drawing of the PET-CNC TENG device developed in this work. (D) Real PET-CNC TENG device (25 mm × 25 mm). (E) Transparent nanocellulose thin film used in the TENG device. (F) Representative electrical output curve obtained for a single contact-separation cycle.

The TENG devices were prepared from an ITO-coated polyethylene terephthalate (PET) sheet (Sigma-Aldrich, UK) and the prepared nanocellulose (CNC) films. The PET tribolayer (thickness 127 μm) comes with a conducting coating of indium tin oxide (resistivity 60 Ω/sq). ITO coating on 4 mm PMMA (backing plate) works as an electrode on the PET tribolayer. For the contact pair of CNC-PET TENG, nanocellulose films were utilized (preparation described in the previous section). Both PET and CNC films were cut out to 25 × 25 mm size (effective nominal area of TENG device). Conducting copper tape (3 M copper foil) was carefully pasted on the bottom side of the nanocellulose tribolayer. The electrode was then insulated with polyimide tape. Both tribolayers (tribo-negative layer of PET and tribo-positive layer of CNC) were connected via copper lead wire to record the electrical response from CNC-PET TENG (Figure 1d). A simplified schematic of the developed TENG device is presented in Figure 1a.

The PET-CNC TENG devices were evaluated in a custom-built Instron Electro-Pulse test rig as described in a previous paper.^37^ The test rig operated in the contact separation mode. As the contact was formed between two nominally smooth surfaces, it was important to obtain a perfectly parallel contact. To achieve this, the test rig was equipped with a contact alignment system. Before starting each measurement, the 360° rotation stage was adjusted to achieve the near-perfect alignment and tightened once aligned. The electromechanical equipment (E3000, Electrodynamic Instron UK) is capable of precisely applying different normal loads and frequencies. The test protocol was programmed to achieve a constant peak-to-peak separation distance of 2 mm. The electrical output performance of the PET-CNC TENG was recorded by using a digital oscilloscope (MSO-X 4154 A, Keysight, USA). The output voltage at each set of load and frequency was measured after a few hundred contact-separation cycles during the stable working stage. Electromechanical measurements were carried out to investigate the effect of the normal load at two different oscillating frequencies. All of the measurements were carried out in a climate-controlled room (20 °C at 50% RH).

## 4 Results and Discussion

### 4.1 FTIR Spectroscopy and Thermal Analysis of CNC and Modified CNC Films

**Figure 2:**
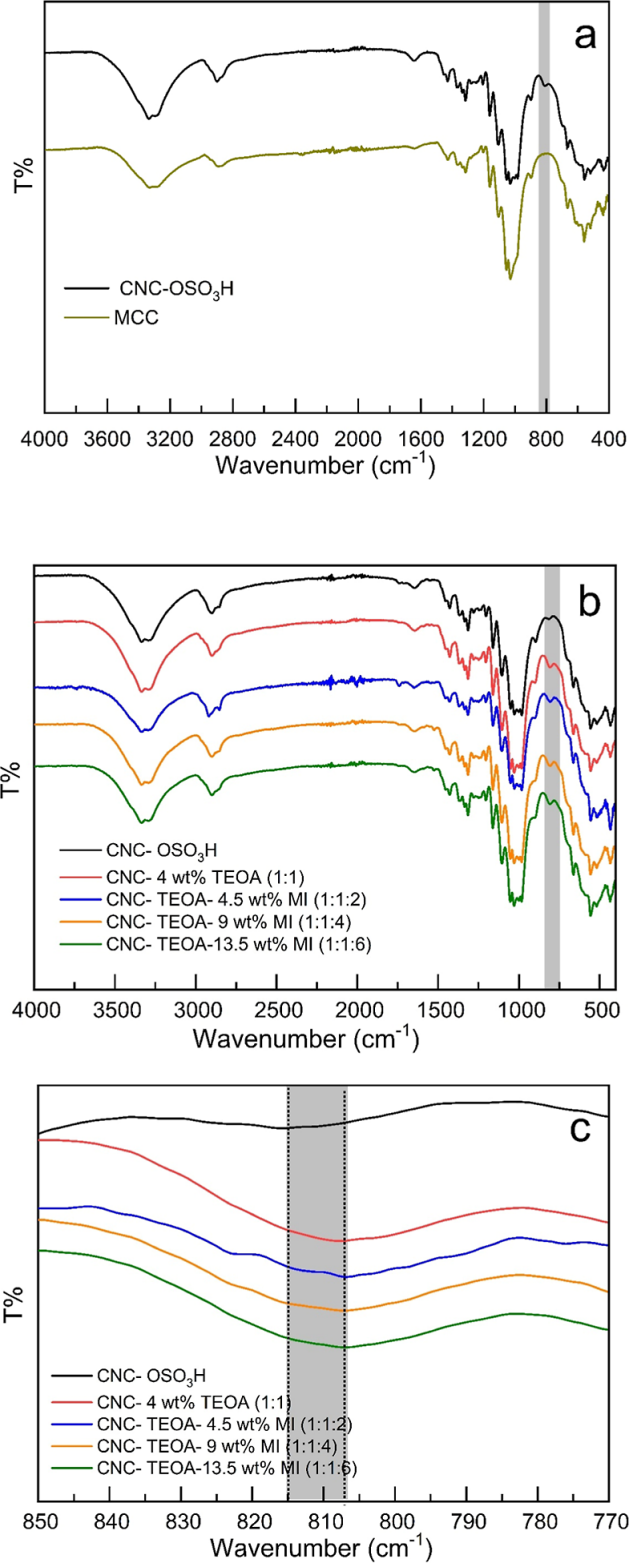
(a) FTIR spectra of MCC and CNC powder and CNC film, (b) FTIR spectra from films of CNC and different mol ratios of CNC-TEOA-MI films, and (c) expansion of the region of sulfate signal in the same samples as of b.

**Figure 3:**
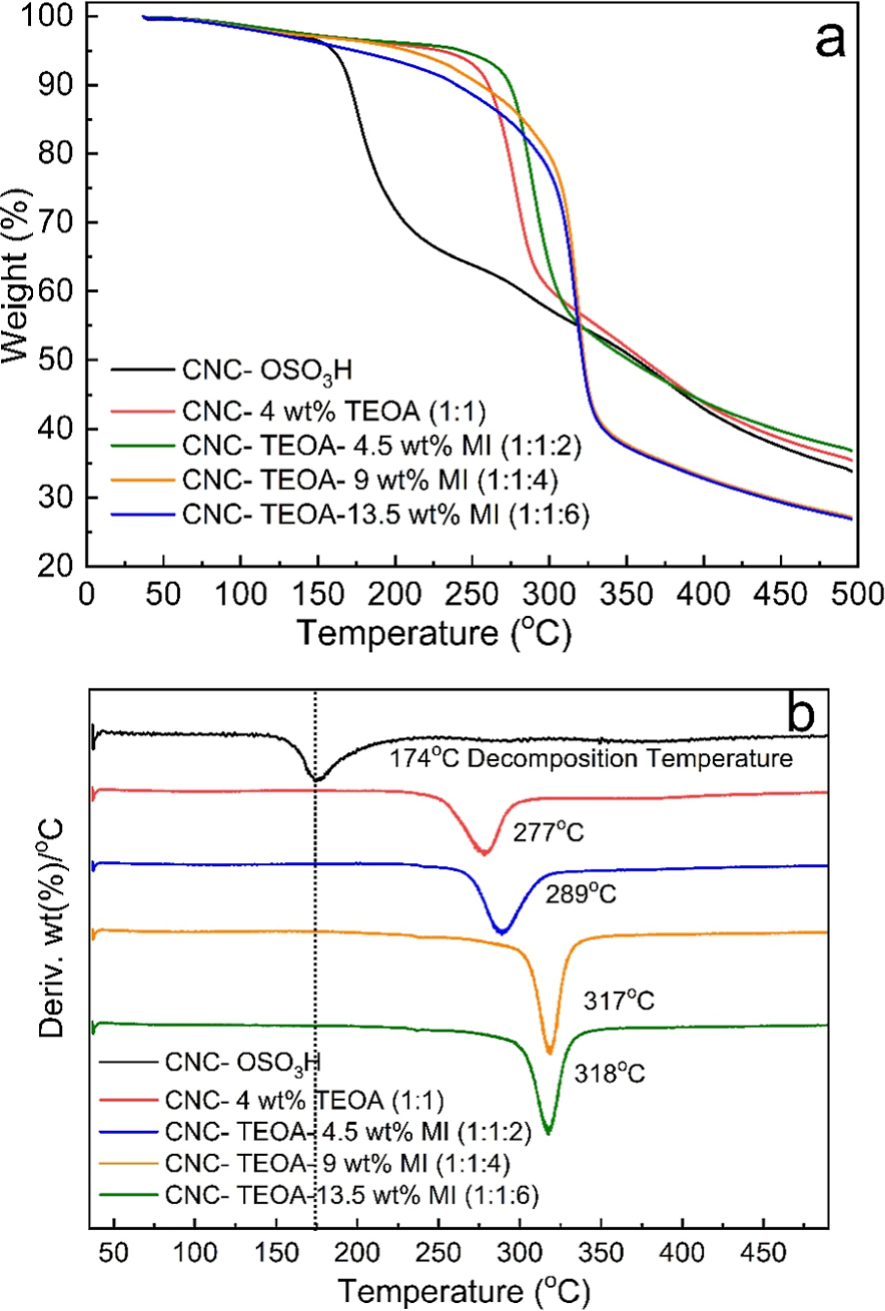
(a) TGA and (b) DTG of CNC, CNC-TEOA (1:1), CNC-TEOA-MI (1:1:2), CNC-TEOA-MI (1:1:4), and CNC-TEOA-MI (1:1:6) samples.

As a reference sample, MCC was used to achieve a typical FTIR on cellulose (Figure 2a). The MCC exhibited the expected signals corresponding to O–H stretching (3300 cm^–1^) caused by inter- and intramolecular vibrations of hydroxyl groups bonded by H-bonds, aliphatic saturated C–H stretching in glucose units (2900 cm^–1^), water bending mode (1640 cm^–1^), and glycosidic bond linkage (1080 cm^–1^).^36,38^ In the case of cellulose nanocrystals (Figure 2b), the FTIR spectra showed, in addition to the peaks observed in MCC, an additional signal at 814–812 cm^–1^ (gray area in Figure 2b) originating from half sulfate esters (−C_6_–O–SO_3_H) or other C–O–S bonds on the CNC surfaces.^38−40^ Furthermore, there was an increase in intensity and a slight shift in frequency for sulfate groups (gray area) in samples with TEOA and MI, confirming that TEOA coordinates with the sulfate groups. TGA analysis provided additional evidence of the TEOA-sulfate interaction. In samples containing TEOA, the thermal decomposition temperature increased by almost 150° (Figure 3b). This is due to the fact that TEOA forms an ion complex with the sulfate groups and thereby blocks the acid-catalyzed degradation,^41^ as described in the previous reports^13,42^ (Figure 3a,b). Since the additives are added in extremely small amounts compared to the glucose units in cellulose, there are no clear signals from them.

In detail, the thermal stability of CNC films derived from MCC and modified CNC films is represented by TGA (Figure 3a). Additionally, the first derivatives of thermal degradation are plotted as differential thermogravimetric analysis (DTGA) in Figure 3b to analyze the maximum degradation temperature. The change in thermal stability is observed and explained in this section, as CNC is prepared from MCC and then modified with different additives; three stages of thermal degradation are observed in all the figures, including in the Supporting Information [see Figure S8a,b]. In the first stage, between 30 and 120 °C, weight loss is primarily due to moisture removal in the samples. The second step occurs at 120–375 °C, where weight loss corresponds to depolymerization, dehydration, and decomposition of glycosyl units. In the third and largest stage, from 375 to 500 °C, weight loss corresponds to the formation of char and gaseous products.^43^ Microcrystalline samples exhibit the highest thermal stability with onset temperatures of 290 and 338 °C for decomposition at the end (Figure S8a,b, in Supporting Information). In the last stage of thermal degradation, the remaining weight is less than 10 wt %, significantly lower than the other samples. For CNC samples without additives, the first two stages of thermal degradation show a significant decrease in thermal stability compared with the MCC samples. This is due to the presence of acidic protons on half sulfate esters, which catalyze thermal degradation through dehydration. These results align with the results of Roman and Winter 2004^42^ and Kim et al. 2001.^44^ In samples containing TEOA, the thermal decomposition temperature increased by almost 150 °C (Figure 3b). The effect is due to TEOA forming an ion complex with sulfate groups, which removes the proton from the sulfate group. By doing so, it inhibits acid-catalyzed degradation,^41^ as described in previous reports^13,42^ (Figure 3a,b).

Based on the FTIR and thermal properties, it can be concluded that GLY does not coordinate with the sulfate groups, whereas TEOA coordinates with the sulfate groups (Figure S8c,d in Supporting Information). In samples containing TEOA, there is a noticeable shift in the frequency of the sulfate group in the FTIR spectrum, as well as a significant increase in the thermal decomposition temperature.

### 4.2 Mechanical Properties of CNC and Plasticized (GLY and TEOA) CNC Films

As mentioned earlier, three different additives, GLY, TEOA, and MI, were chosen to enhance the bendability of the CNC films. GLY, a common plasticizer for carbohydrates like starch, xylan, agar, and agarose, can coordinate with the hydroxyl groups of cellulose.^45−49^ Typically, GLY is added in amounts of 20–45 wt % to increase elongation, but this also significantly reduces the tensile strength. Since sulfated CNC was used in this study, both GLY, which is expected to coordinate with the hydroxyl groups, and TEOA, which is expected to coordinate with the sulfate groups on CNC and form hydrogen bonds with the hydroxyl groups on the CNC surface, were investigated to improve the elongation property of the CNC films. Initially, three different concentrations, 10, 20, and 25 wt % of GLY or TEOA, were used. These ratios were chosen to correspond to the amount of GLY commonly used to achieve the good plasticization of polysaccharides. It is important to note that the suspensions and films containing TEOA have a neutral pH, while the films containing GLY remain acidic. The mechanical properties of the CNC and CNC-additive films are summarized in Table S3 (for GLY and TEOA-CNC films in Supporting Information) and Table 1 in terms of tensile strength (MPa) and elongation at break (%). As seen from the tensile data, neither of the two additives improves the elongation of the CNC films but results in a significant decrease in the tensile strength. Compared to other carbohydrates where GLY provides significant elongation, the main difference is that many of the carbohydrates are flexible and can be aggregated through intra- and intermolecular hydrogen bonds. When an additive such as GLY is added, these hydrogen bonds may be disrupted, resulting in increased elongation as a bulk effect. However, in the case of CNC, which is rigid and has a robust structure, the additives can only bind to the surface and will affect only the interaction between crystallites, not the inherent stiffness of the crystallites. Therefore, the plasticizer acts more like a lubricant between crystallite surfaces. As observed from the results, the addition of GLY significantly reduced the tensile strength. This implies that GLY reduces the interaction between CNC crystallites, thereby keeping them apart. For the films containing TEOA , the elongation is slightly higher and the tensile strength is also slightly higher.

**Table 1:** Tensile Properties of CNC and Modified CNC Films^a^

sample no.	CNC films	tensile strength (MPa)	elongation at break (%)
1	CNC–OSO_3_H (sulfated)	47 ± 3.2^b^	1.3 ± 0.5
2	CNC-TEOA (1:0.6) 2.5 wt %	67 ± 1.8	2.72 ± 0.3
3	CNC-TEOA (1:1) 4 wt %	65.7 ± 3.8	3.08 ± 0.3
4	CNC-TEOA (1:1.2) 5 wt %	55.5 ± 1.7	2.1 ± 0.6
5	CNC-TEOA (1:1.8) 7.5 wt %	50.8 ± 1.9	2.8 ± 0.35
6	CNC-TEOA (1:2.4) 10 wt %	48.5 ± 3.9	3.5 ± 0.22
7	CNC-TEOA (1:3) 12.5 wt %	37.8 ± 2.9	2.8 ± 0.6
8	CNC-TEOA (1:3.6) 15 wt %	33.4 ± 1.5	3.38 ± 0.24
9	CNC-TEOA (1:4.2) 17.5 wt %	26.2 ± 1.6	2.6 ± 0.24
10	CNC-TEOA (1:4.8) 20 wt %	20.1 ± 2.4	3.4 ± 0.26
11	CNC-TEOA (1:5.4) 22.5 wt %	20 ± 1.5	3.5 ± 0.28
12	CNC-TEOA (1:6) 25 wt %	15.7 ± 0.8	2.6 ± 0.3
13	CNC-TEOA-(MI) (1:1:2)	68.6 ± 3	4.83 ± 0.2
14	CNC-TEOA-MI (1:1:4)	52 ± 1.6	3.6 ± 0.2
15	CNC-TEOA-MI (1:1:6)	38.4 ± 4.1	2.5 ± 0.18

a For samples 13, 14, and 15, in terms of wt %, these concentrations are equal to CNC: 4 wt %TEOA:4.5 wt % MI (1:1:2), CNC: 4 wt %TEOA: 9 wt % MI (1:1:4), CNC:4 wt % TEOA:13.5 wt %MI (1:1:6).

b Standard error of the mean values.

From Table S3 (Supporting Information), it was observed and concluded that the plasticizer did not enhance elongation in CNC films, as it was less than 5%. However, films containing GLY or TEOA were bendable and did not exhibit any cracks when bent repeatedly with fingers. Therefore, these films possessed properties suitable for TENG applications. Before conducting the TENG evaluation, we decided to further optimize the amount of TEOA added to CNC. This time, our focus was to add just enough TEOA to occupy the sulfate groups, aiming for a slight improvement in elongation without compromising tensile strength.

**Figure 4:**
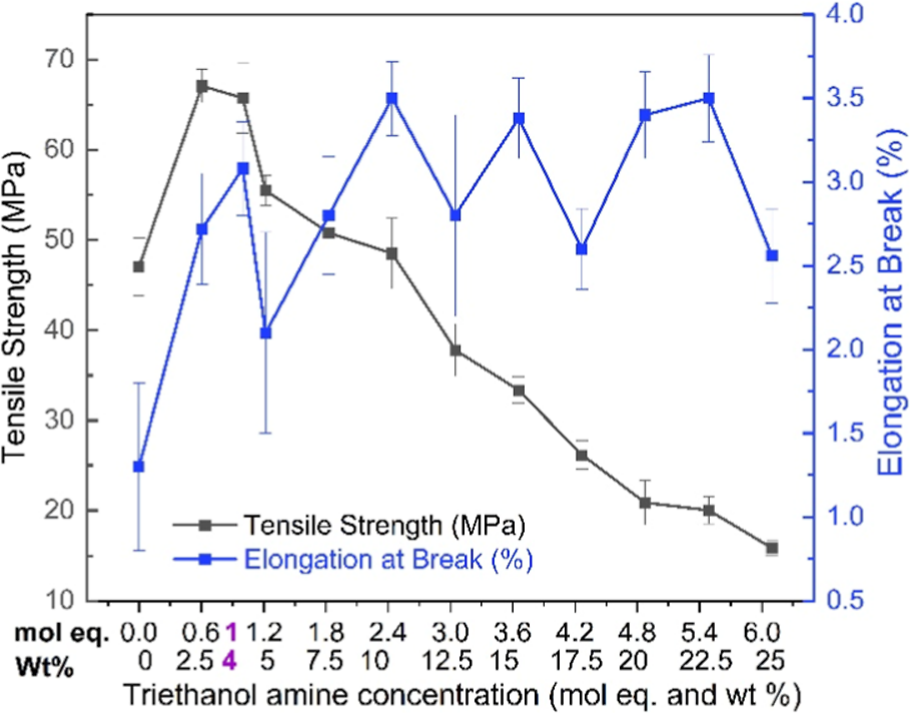
Tensile properties of CNC and TEOA-CNC films with different concentrations. In the *x*-axis, mentioned concentrations (1 mol equivalent equals to 4 wt %) between 0.6 (2.5 wt %) and 1.2 (5 wt %) is the optimum concentration.

To optimize the effect of TEOA, an evaluation was performed on the molar amount of TEOA relative to the sulfate content on the surfaces of CNC. The evaluation included concentrations ranging from 0.6 to 6 mol equivalents of TEOA relative to the sulfate content, with intervals of 0.6 mol equivalents. As many materials chemists use weight % when making blends, the TEOA concentration is also expressed in weight percentage (wt %) 0.6 to 6 mol equivalents of TEOA is equal to 2.5 to 25 wt % with intervals of 2.5 wt.%. Table 1 presents the data for CNC and TEOA plasticized CNC films showing different concentrations of TEOA. Figure 4 and Table 1 reveal that when the molar amount of TEOA exceeds the sulfate concentration, there is a clear decrease in tensile strength. However, there is no significant increase in elongation. Therefore, we decided to use 1 mol equivalent to maintain high tensile strength and achieve some elongation.

To further improve the elongation, MI was introduced as an additional additive. This choice was based on a previous study, where we demonstrated that adding a small amount of MI to pulp fibers significantly enhanced the rate of pulp dissolution in the ionic liquid, EMIMAc.^50^ With the addition of MI, the pulp was completely dissolved in just a few minutes. MI was added to 4 wt % TEOA-CNC films at concentrations of 2 mol equiv (4.5 wt %), 4 mol equiv (9 wt %), and 6 mol equiv (13.5 wt %) with respect to the sulfate groups. The best material characteristics were achieved with 2 mol equiv of MI, although the effect on tensile strength was not significant upon addition of MI. The tensile strength increased from 66 to 68 MPa, and the elongation increased from 3 to 4.8%. The value of elongation at break is higher than the previous reports on pure CNC films^30,31^ that were prepared without blending CNC with CNF or other cellulose derivatives to improve this property.

The selective coordination of MI to the axial –H groups is implied by a shift in 2θ value for 200 planes to lower theta value in XRD analysis for CNC-TEOA-MI samples (see Supporting Information, Figure S1 and Table S1) and further supported by reports on the nonpolar hydrogen-p interaction.^51,52^

### 4.3 Electromechanical Performance of PET-CNC TENGs

**Figure 5:**
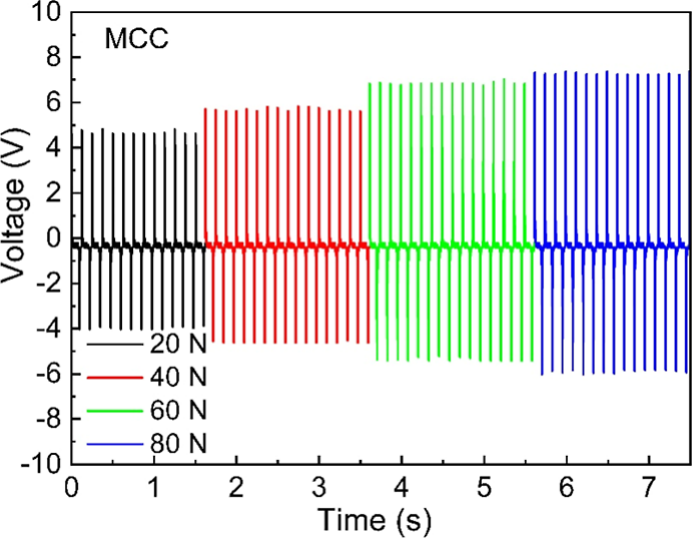
Electrical characteristics (output voltage) of the TENG device fabricated with the MCC sample with increasing contact force and at an oscillating frequency of 8 Hz.

**Figure 6:**
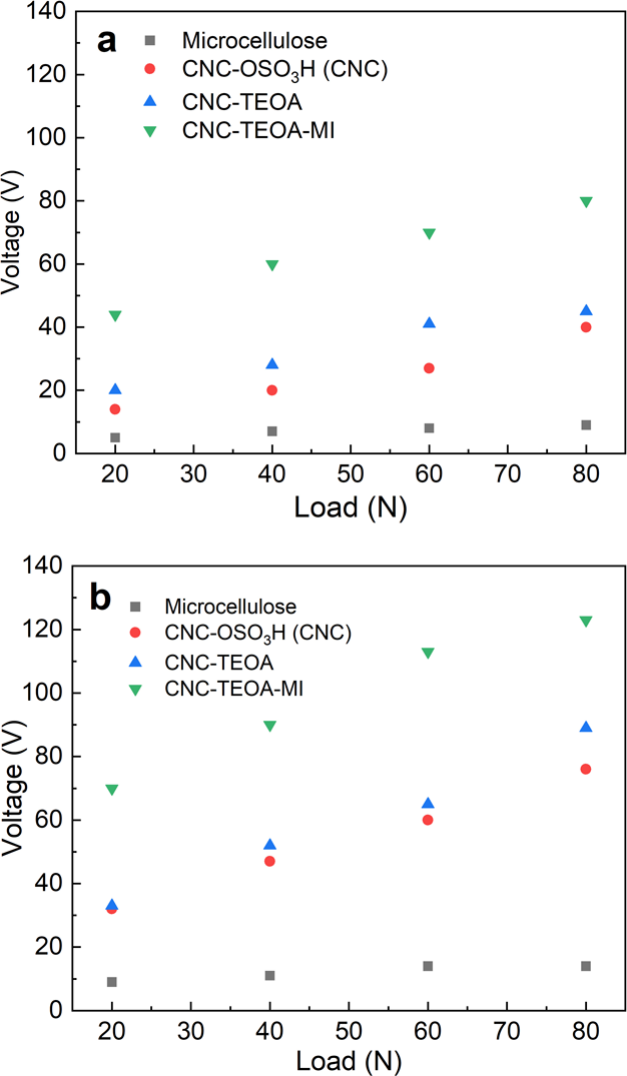
Effect of contact force on the electrical performance (output voltage) of all four PET-CNC TENG devices at two frequencies: (a) at 4 Hz and (b) at 8 Hz.

The triboelectric response of the PET-CNC TENG was systematically investigated by varying the normal force at 20, 40, 60, and 80 N. A contact force of 20 N corresponds to a nominal contact pressure of 32 kPa. Additionally, each set of measurements was carried out at two frequencies: 4 and 8 Hz. Figure 5 shows that the output voltage signal is uniform and continuous for all four contact forces investigated for MCC films. The operational principle of CNT-PET TENG is illustrated schematically in Figure S10 of the Supporting Information. It is confirming the precise control of the contact-separation process and the high stability of the TENG operation. Figure 6a,b demonstrates that all four TENG devices produce a higher output voltage at an oscillating frequency of 8 Hz compared to 4 Hz (see Figure S11 in Supporting Information). The increase in electrical output at a higher frequency can be attributed to a shorter contact time and an increased rate of change of potential and capacitance between the electrodes.^53^ It can be observed that MCC has the lowest output voltage, while the film with both TEOA and MI exhibits the highest output voltage output. At the maximum contact force (*F*_n_ = 80 N) and 8 Hz oscillating frequency, the CNC-TEOA-MI demonstrates the highest output voltage (*V*_out_ ≈ 123 V), followed by CNC-TEOA (*V*_out_ ≈ 89 V), CNC–OSO_3_H (*V*_out_ ≈ 76 V), and microcellulose (*V*_out_ ≈ 14 V) (Figure 6b).

The explanation may be that MCC has the lowest crystallinity, and all crystallites have a random orientation, thus showing the lowest electrical performance. On the other hand, CNC has a high order of cellulose chains, resulting in a more uniform dipole moment and better electrical performance.^54−56^ However, the nanocellulose samples have nonuniformly ordered crystallites but still retain structural organization. In addition, in a diluted suspension, CNC crystallites rotate randomly. As evaporation increases concentration, crystallites interact through their DLVO layer, forming bundles without a uniform direction. These bundles aggregate, sediment, and align uniaxially along the Petri dish surface. This alignment is less pronounced for MCC particles due to their square shape and lower aspect ratio.^57,58^ Interestingly, the films containing TEOA have higher voltage output than pristine sulfated CNC. This may be due to an increase in roughness and dielectric constant with a higher degree of crystallite order and/or slightly higher flexibility/moveability between the crystallites when TEOA and MI are present in the films (see Figure S12 and Table S4 Supporting Information). This allows the crystallites to move into charge-separated structures, tearing apart the sulfate ammonium complex upon tapping. From analysis of the polarized optical microscopy (POM) pictures (see Supporting Information, Figures S2–S6, Table S2), it is difficult to determine a significant difference in the organization of crystallites in the films. Another possibility for the higher electrical properties of the CNC-TEOA-MI TENG may be due to the excellent proton and electron transport properties of imidazole derivatives.^59^

In the absence of dedicated characterization to verify the alignment in CNC, it is believed that TEOA and MI replace the dense inter-CNC hydrogen bonding network with a looser one, facilitating charge transport. The improved performance of the TENG device with CNC compared to MCC is due to the presence of sulfate groups, reduction in dimensions, and removal of amorphous regions during hydrolysis. Sulfuric acid hydrolysis increases the dielectric constant of CNC due to hydrogen bonding and stronger interfacial polarization.^60,61^ MCC tends to have larger, unevenly sized particles due to lower surface charge density compared to CNC. Smaller, higher aspect ratio CNC particles result in more surface dipoles per volume, giving a higher dielectric constant. Sulfate groups also improve the proton transfer ability and hydrophilicity, increasing charge carrier mobility. CNC aggregates slower than MCC during drying, leading to better-aligned crystallites and fewer defects,^13^ improving electromechanical performance. The high crystallinity index for all samples suggests that sulfuric acid may partly dissolve crystalline cellulose,^62^ removing accessible amorphous regions and reacting with crystalline surface regions, resulting in similar crystallinity but more ordered CNC surfaces, enhancing long-range intracrystallite charge transport.

For a comprehensive comparison, the peak-to-peak voltage outputs for all four devices are shown in Figure 6. At the lowest contact force (*F*_n_ = 20 N), the Mica CNC-TEOA-MI TENG exhibited the highest value (*V*_out_ ≈ 44 V). It is worth noting that there are clear increases in output voltages for all four TENGs with an increase in the normal contact force. This contact force-dependent behavior can be explained by an increase in the real contact area as the force increases, as reported in previous literature.^37,63^ In the case of the PET-CNC-TEOA-MI TENG, the output voltage increases from *V*_out_ ≈ 44 V at 20 N to *V*_out_ ≈ 80 V at 80 N (Figure 6a). However, the rate of increment decreases for the PET- Microcellulose case (*V*_out_ ≈ 5 V at 20 N to *V*_out_ ≈ 9 V at 80 N). Oscillating frequency is also an important parameter to investigate.

**Figure 7:**
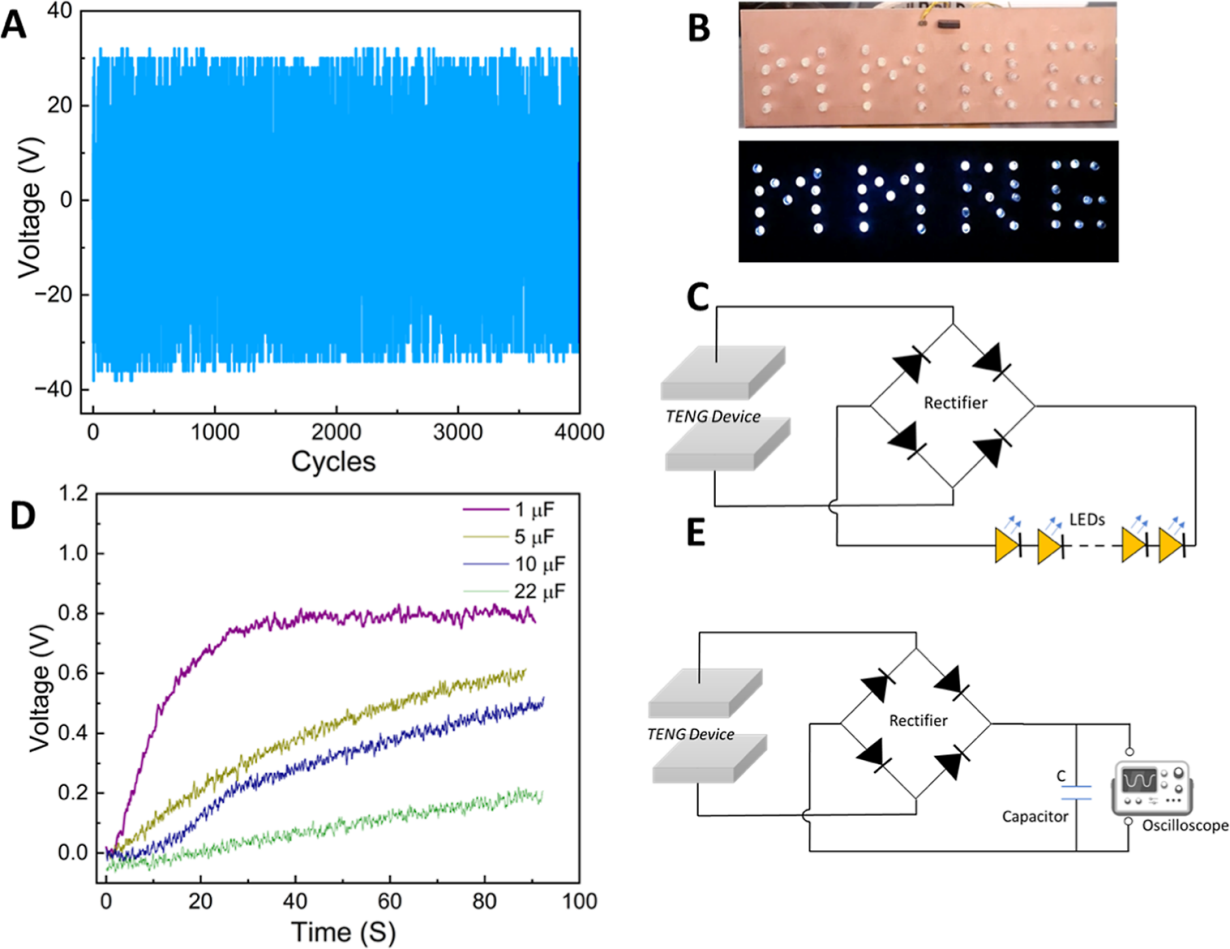
(A) Long-term stability performance of optimized PET-CNC TENG (CNC-TEOA-MI). The stability test was carried out at the normal load of 40 N and the oscillating frequency of 4 Hz. (B) White LEDs representing the “MMRG” logo are illuminated from the power generated from the PET-CNC TENG device (see video, SV1 in Supporting Information). (C) Circuit diagram for LEDs powered by CNC-PET TENG. (D) Charging characteristics of four different capacitors charged by the CNC-PET TENG. (E) Circuit diagram for the capacitor charging measurements.

For energy harvesting applications, it is important to investigate the stability and endurance of an energy harvesting device. To examine this, the optimized TENG device (PET-CNC-TEOA-MI) was operated at a normal load of 40 N and an oscillating frequency of 4 Hz. Figure 7a confirms stable and continuous power generation (output voltage ≈60 V) for about 4000 contact-separation cycles. Even after running for long numbers of cycles, we did not observe any visible wear and tear on both of the tribo-layers. Furthermore, to demonstrate the promising practical ability of power produced from PET-CNC TENG, an array of 40 white LEDs was illuminated (Figure 7b). Toward the practical application, we have demonstrated the developed TENG device’s ability to store generated power in a capacitor (Figure 7D). For this, four different capacitors with capacitance ranging from 1 to 22 μF were tested. The stored power could potentially be used for powering small electronic devices.

It is acknowledged that varying material types, working conditions, and areas for each TENG make direct comparisons challenging. Table 2 summarizes the output voltages of various cellulose-based TENGs, including our own. Differences in the TENG type, cellulose-polymer composites, and working conditions can influence performance. Our TENG, with a maximum output voltage of 80 to 123 V, demonstrates competitive performance among cellulose tribolayers in TENG (Table 2).

**Table 2:** Comparison of CNC-PET TENG with Earlier Published Reports on Cellulose-Based TENG

materials	output vol (V_out_)	title of the work	reports
cellulose nanocrystals (CNC)	10.01 V	cellulose-based triboelectric nanogenerator prepared by multi-fluid electrospinning for respiratory protection and self-powered sensing	(29)
nanocrystalline cellulose (NCC)	2.04 V	energy harvesting through the triboelectric nanogenerator (TENG) based on polyurethane/cellulose nanocrystal	(28)
CNC	400 V	PDMS composite generator film impregnated with CNC for enhanced triboelectric performance	(27)
91% CNC-PET	123 V	bendable substrates of cellulose nanocrystals for triboelectric nanogenerators	this work
cellulose nanofibrils (CNFs)	94 V	all-cellulose nanofiber-based sustainable triboelectric nanogenerators for enhanced energy harvesting	(64)
cellulose nanofibrils (CNFs)	205 V	low-cost, environmentally friendly, and high-performance cellulose-based triboelectric nanogenerator for self-powered human motion monitoring	(65)

## 5 Conclusions

This work successfully demonstrated the applicability of cellulose nanocrystal (CNC)-based bendable substrates as a positive tribolayer in the development of TENG (Triboelectric nanogenerators) with PET (negative tribolayer). The bendability of CNCs is improved by the addition of TEOA and MI. TEOA coordinates with sulfate groups on the CNCs planes (110) and (1–10), while MI adsorbs to the hydrophobic plane (200). A 1:1 mol equiv of plasticizer (TEOA) was found to provide the best mechanical properties (strength and elongation % at break). Further, with the addition of MI, the poor elongation at break for pristine CNC was increased to ∼approximately 5% with a strength of 68 MPa. The total amount of additives used is less than 10 wt %, thus the CNC content is more than 90 wt %, which CNC films including TEOA and MI (CNC-TEOA-MI films) show the best electromechanical performance. Under the conditions of a maximum contact force of 80 N and an oscillating frequency of 8 Hz, CNC-TEOA-MI exhibited the highest output voltage, approximately 123 V. This was followed by CNC-TEOA with an output voltage of around 89 V, CNC–OSO_3_H at about 76 V, and Micro crystalline cellulose at roughly 14 V. The TENG results of cellulose samples, including MCC, and CNC samples with and without additives are rationalized in terms of the surface to volume of dipoles, the intrinsic polar and proton and/or electron transport properties of surface groups and additives, the charge carrier mobility, and the intercrystallite distance and alignment. The TENG results suggest potential applications in self-powered sensors and biomedical devices, enhancing sustainability and functionality. Considering the different outputs from these electromechanical results also helps in characterizing the type of cellulose samples: crystalline, semicrystalline, and amorphous.

## Supporting Information Available

White LEDs representing the “MMRG” logo illuminated from the power generated from the PET-CNC TENG device (MP4)
Crystallinity by XRD, POM, UV–vis spectroscopy, wettability, TGA, and tensile results (PDF)
