# Supplementary material for: Bendable Substrates of Cellulose Nanocrystals for Triboelectric Nanogenerators
Source: ACS Appl Nano Mater. 2025 May 3;8(19):9868–77. doi: 10.1021/acsanm.5c01087 (PMC12090183; doi:10.1021/acsanm.5c01087)
Supplement: Supplementary file 2 — an5c01087_si_002.pdf [file an5c01087_si_002.pdf]

Supporting Information

Bendable Substrates of Cellulose Nanocrystals for Triboelectric Nanogenerators

Amit Kumar Sonker[αβξ*], Charchit Kumar[¥], Hannah Tideland[ζ], Satyaranjan Bairagi[¥λ], Nirmal Kumar Katiyar[±], Daniel M. Mulvihill[¥], Gunnar Westman[βξ*]

[α]BA5409, Cellulose films and coatings, BA54 Biomaterials Processing and Products, VTT Technical Research Centre of Finland, Tietotie 4E, Espoo, 02150, Finland

[β]Department of Chemistry and Chemical Engineering, Chalmers University of Technology, Gothenberg, 41296, Sweden

[ζ]Department of Chemistry and Molecular Biology, University of Gothenburg, Gothenberg-40530, Sweden

[±]Department of Chemistry, Amity institute of applied Science, Amity University. Noida- 201313, Uttar Pradesh, India

[ξ]Wallenberg Wood Science Centre, Chalmers University of Technology, Gothenburg, 41296, Sweden

[¥] Materials and Manufacturing Research Group, James Watt School of Engineering, University of Glasgow, Glasgow, G12 Q88, UK.

[λ]Biomedical and Mobile Health Technology Lab, Department of Health Science and Technology, ETH Zurich. Lengghalde 5, 80008, Zurich, Switzerland

Corresponding authors email –amit.sonker@vtt.fi, westman@chalmers.se

S1. X-Ray Diffraction (XRD) – Crystallinity and crystallize size.

XRD measurements were performed on a Bruker D8 Advance instrument in the range of 10-50° using scan rate 0.5sec/step. The measurements were carried using Cu K-a - 1.54 Å, and samples were mounted on Silicon single crystal sample holder. The crystallinity Index of cellulose samples were measured by equation as described by Daicho et al. [1]

$$Crystallinity\ Index\ (CI) = Area_{cryst} / Area_{Total} \times 100 \qquad \textbf{(Eq 1)}$$

Whereas $Area_{cryst}$ is total areas under peaks for crystalline regions and $Area_{Total}$ is total area under peaks for crystalline and amorphous regions.

Cellulose nanocrystals (CNC) are obtained by acid hydrolysis of microcrystalline cellulose (MCC) and expected to be crystalline, free from amorphous regions compared to MCC sample and therefore, crystallinity of cellulose samples is measured by X-ray diffraction (XRD) **(figure S1)**. Microcrystalline cellulose (5a) has characteristic peaks at 2θ - 14.6° (1-10 plane), 16.4° (110), 20.6° (012), 22.6° (200), and 34.5° (004), and the peak areas are used to calculate crystallinity index in percentage as described by Daicho et al [1]. The effect of plasticizer addition on crystallinity of CNC films is considered. Introducing ductility in semicrystalline polymeric system is expected to decrease the crystallinity of polymers. Here, in pure crystalline cellulose system, Microcrystalline and nanocrystalline system, the measured crystallinity values are 90

and 96% [2]. For cellulose nanocrystals, the observed value of CI is higher as it is free from amorphous region of cellulose chains. For all, film samples crystallinity index was measured by considering only four peaks at 2θ - 14.6° (1-10 plane), 16.4° (110), 20.6° (012), and 22.6° (200).

The introduction of TEOA, on hydrophilic surfaces of CNC and MI on hydrophobic surfaces did not change the crystallinity significantly **(Table S1)** but there is peak shift in 2θ values for different cellulose samples was observed. Comparing Microcrystalline cellulose and powdered samples, the peak at 22.6° corresponding to 200 shifting to higher angle values which indicates expansion of the H-bonded cellulose chains. And considering CNC samples, the same peak for 200 planes is shifting to lower angle values which confirmed contraction of H-bonded planes in cellulose structures. Further, the change in peak shift for other planes (110 and 1-10) is not clearly visible in the diffract graphs, and therefore deconvolution is performed to see the shift in the peak. Also, the expansion and contraction of H-bonded polymer chains is correlated and supported by the increase or decrease in crystallite size (CS) corresponding to all peaks (Table S1). Further, the change in peak shift for other planes (110 and 1-10) is not significant as shown in figure S1, and therefore deconvolution is performed to see the shift in the peak. The expansion and contraction of H-bonded polymer chains are correlated and supported by the increase or decrease in crystallite size (CS) corresponding to all peaks [3]. This relationship can be explained by the principle that changes in the spacing between atomic planes in a crystal, which affect the diffraction pattern, are indicative of changes in the crystallite size and the arrangement of polymer chains.

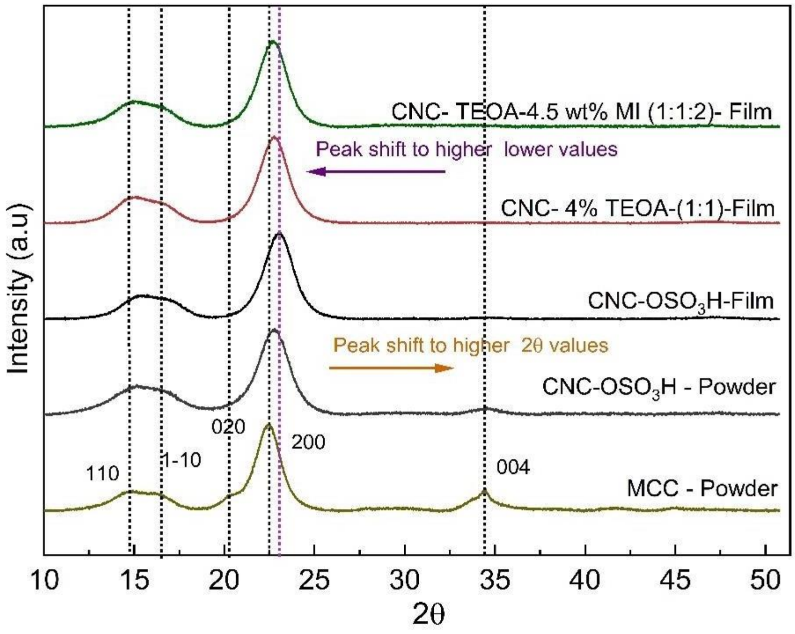

Figure S1 - XRD plots of MCC, CNC and modified CNC samples

**Samples**	**Crystallinity Index (CI) (%)**	**C ystallite size (CS) in nm**				
**CNC films**	CI	C S_{1-10} (nm)	C S_{110} (nm)	CS_{012} (nm)	C S_{200} (nm)	C S_{004} (nm)
**CNC- 4 wt%-TEOA-4.5 wt% (MI) (1:1:2) - Film**	90%	4.4	4.8	--	3.3	--
**CNC-4 wt %- TEOA (1:1:)-Film**	92%	4	4.9	--	3.1	--
**CNC- OSO_3H -Film**	96%	3.8	4.6	--	3.3	--
**CNC-OSO_3H-powder**	94%	3	4.2	--	3.4	5.6
**MCC-powder**	90%	3.5	5.7	6.2	6.3	5.7

Table S1 - Crystallinity percentage and crystallite size of MCC, CNC and modified CNC films

In terms of wt%, these concentrations are equal to CNC OSO_3H: TEOA:MI - 4.5wt%, CNC-OSO_3H: TEOA4wt%:MI - 9 wt%, CNC-OSO_3H: TEOA4wt%:MI - 13.5wt%

S2. Polarized optical microscopy of CNC films.

CNC films were examined using polarized optical microscopy (POM) using a CarlZeiss Axio Scope. A1 (Oberkochen, Germany) in brightfield mode using linear polarizers. The magnification was 100x after initial inspection with a 40x lens. The selected resolution gave fields of view of approximately 25×20 nm. POM images using cross-polarized light were taken to estimate the birefringence and microstructure of the films. Varying interference colors across the micrographs and films indicate that films are birefringent with a multi-grain structure resulting from the varying orientations of CNCs. The polycrystalline structure is well-documented for CNC films dried at ambient conditions without external alignment such as magnetic field or shear due to kinetic arrest of the suspension occurring long before thermodynamic equilibrium is reached, as well as processes such as capillary flow.[4] Nevertheless, a relatively large grain size and flat structure on the microscale can be observed in **figure S2 and S3**, which has been shown to correlate strongly with relatively defect-free film with closer packing, high tensile strength, elongation at break, and transparency,[5] (**figure S4**). Using an updated version of the Michel-Lévy interference color chart[6] retardation wavelength and birefringence of films were estimated (**Table S2**). The CNC-OSO3H TEOA (1:1) and CNC-OSO3H TEOA-MI (1:1:2) films (56.4±2.9 and 46.3±2.3 µm respectively) were estimated to have birefringence values of 0.011-0.015 and 0.014-0.020 respectively, in reasonable agreement with the values in the literature of 0.021 and 0.026, for dip-coated CNC films of two different thicknesses.[7] Estimates for CNC-OSO_3H and CNC-OSO_3Et_4N films (**figure S5** and **Table S2**) can only be regarded as rough because these films were significantly thicker than the range of the Michel-Lévy color chart, (50 µm, films>70 µm). Nevertheless, these estimates are of the same order of magnitude of birefringence as the thinner films with added plasticizers.

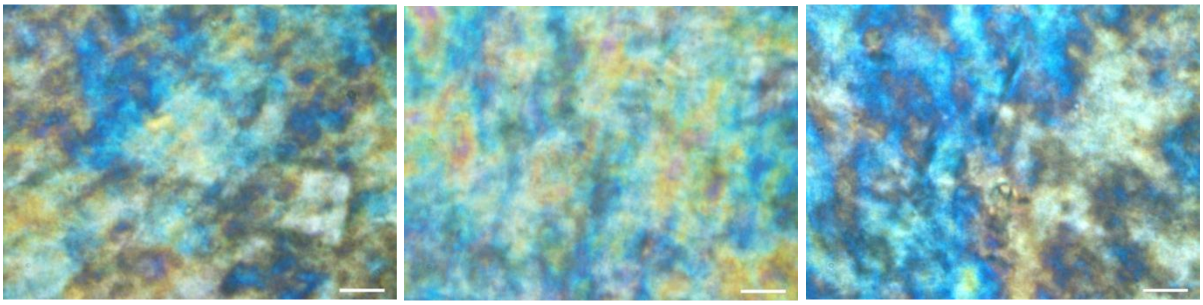

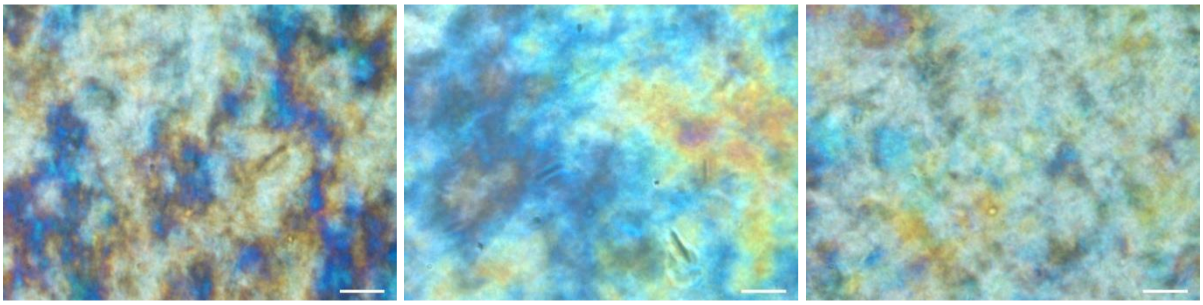

Figure S2 - POM micrographs of CNC films with TEOA:OSO_3H in a 1:1 molar ratio. Scale bars: 5 µm.

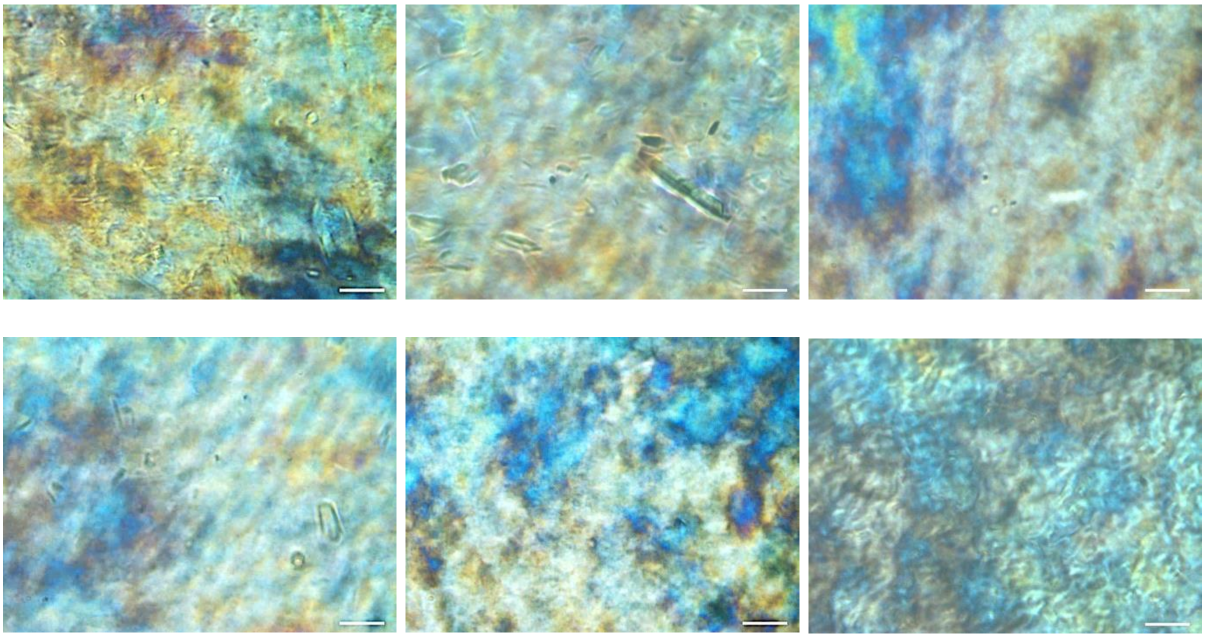

Figure S3 - POM micrographs of CNC films with OSO_3H:TEOA:MI in a 1:1:2 molar ratio. Scale bars: 5 µm.

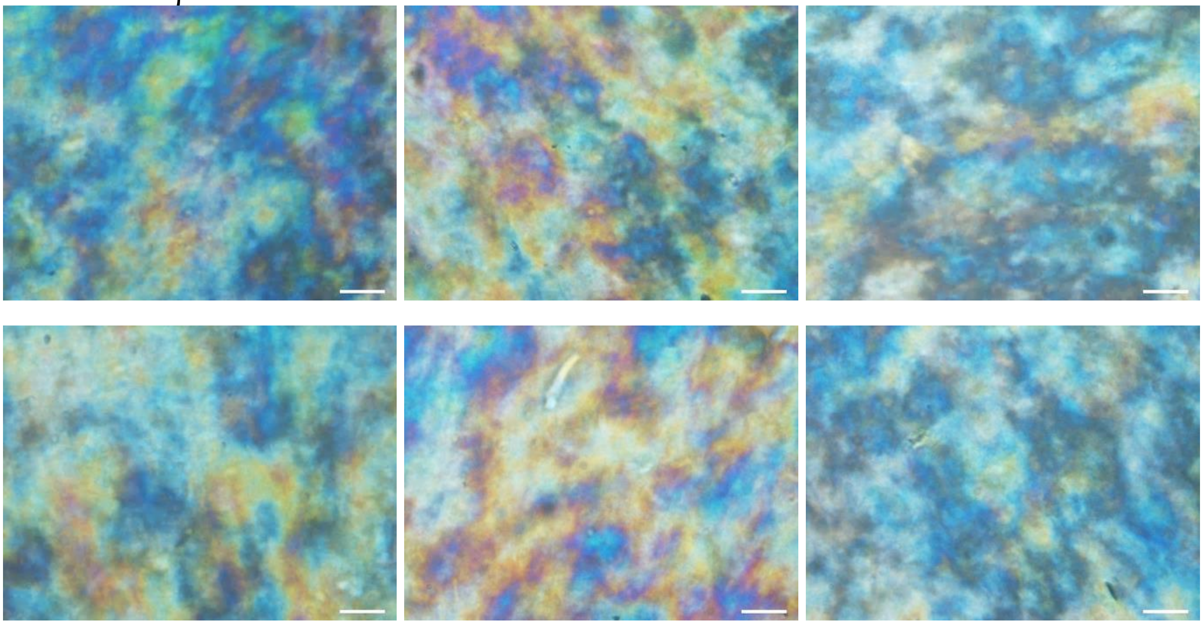

Figure S4 - POM micrographs of CNC-OSO_3H films. Scale bars: 5 µm.

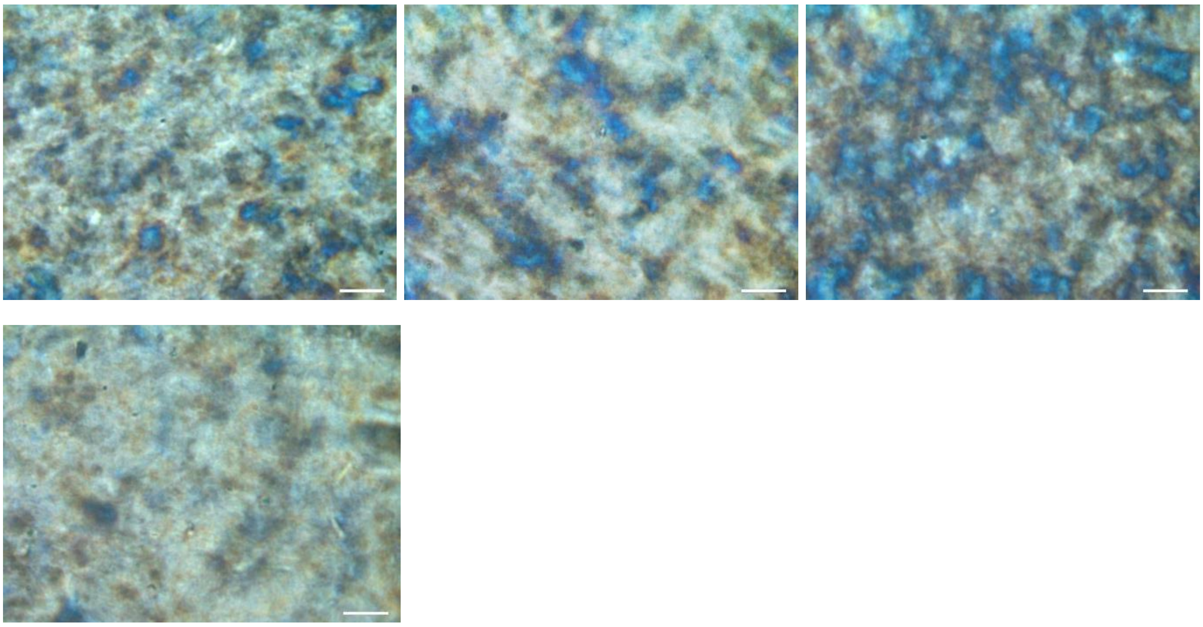

Figure S5 - POM micrographs of CNC-OSO_3Et_4N (CNC-TEOA) (1:1) films. Scale bars: 5 µm.

**Film**	**Birefringence**	**Retardation (nm)**	**Interference colors**	**Mean thickness (µm)**
**CNC-OSO_3H**	0.008-0.020*	600-1200*	2nd order blue to 3rd order turquoise	70 (9.8) *
**CNC- TEOA (1:1)**	0.007-0.021	400-1100	1st order brown to 2nd order purple	56.3 (3)
**CNC-O TEOA- MI (1:1:2)**	0.014-0.023	700-1000	2nd order light blue to pink	46. (2.2)

*Standard error of the mean value

Table S2 - Birefringence-related properties based on POM micrographs (**figure S3-S4**). Retardation of light, Γ, and birefringence, Δn, was estimated for the <60 µm samples based on an updated version of the Michel-Lévy interference color chart [6] and the well-known expression $\Delta n = |n_e - n_o| = \Gamma/d$, where n_e and n_o are the refractive indices of the extraordinary and ordinary rays respectively and d is the thickness. Values marked with asterisk (*) should only be regarded as rough estimates due to the thickness of films being significantly outside the Michel-Lévy chart (50 µm).

S3. UV-vis- spectroscopy

A PerkinElmer Lambda 950 UV-vis spectrophotometer was used to measure UV-vis spectroscopy of solid films at wavelengths from 300 to 800 nm. In the sample holder, each film was placed in the upper part of the window at a 90 ° angle to the incident light. Translucency variations were replicated and averaged across replicates. In order to calculate the attenuation

coefficients for a reflecting medium, assume only one reflection at each interface and neglect the imaginary part of the refractive index as follows.

$T = (1 - R)^2 e^{-\alpha d}$ (Eq 2)

where T is the transmittance of light, $R = (n-1)/(n+1)^2$ is the reflectance, n is the real part of the refractive index, α is the attenuation coefficient and d is the thickness of the film. The real part of the Cauchy dispersion equation for refractive index,[8] was applied using experimental constants and reported by Niskanen et al.,[9] for CNCs in the visible light range of 400–700 nm (in agreement with literary data[10, 11, 12]) and was extrapolated to the range 300-800 nm:

$$n_{eff} = 1.4767 + \frac{0.0063}{\lambda^2} - \frac{0.0002}{\lambda^4}. \qquad \textbf{(Eq 2)}$$

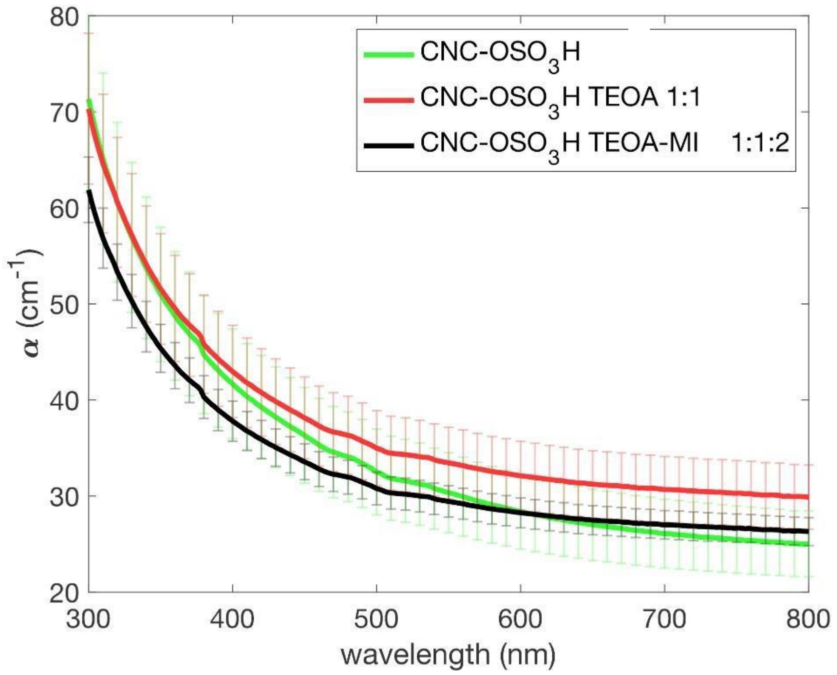

Figure S6 - Attenuation coefficients of CNC film with TEOA:OSO_3H in a 1:1 molar ratio (red) and CMC-TEOA-MI 1:1:2 ratio (black). Error bars indicate the standard deviation. For clarity, every fifth error bar is shown.

The films are highly transparent, with similar attenuation coefficients of 30.4± 4.2 cm^{-1}, 33.5±1.6 cm^{-1} and 29.4±3.7 cm^{-1} for neat CNC-OSO_3H, CNC-OSO_3H-TEOA (1:1) and CNC-OSO_3H-TEOA-MI (1:1:2) films at 550 nm. The light attenuation is comparable to films from

the same source subjected to probe ultrasonication [5], indicating a well-dispersed suspension prior to drying with few larger voids and aggregates in the polycrystalline films, also indicated by the POM micrographs.

S4. Wettability of CNC and modified CNC films

The wettability of CNC films was measured on the surface by measuring contact angles and in the bulk by analyzing water absorption or swelling. An optical goniometer from Attention was used to measure surface wettability and contact angle. The contact angles (**figure S7)** in supplementary files) for CNC and modified CNC samples are measured to see the change in surface water adsorption property of CNC films. Among all CNC films, CNC-OSO_3H films show higher contact angle as with proton as a counter ion the CNC are close in contact with each other due to H-bonding interaction. The packing of crystal is so closed and therefore adsorbed water has no affinity to get adsorbed immediately. On ion exchange, CNC-Triethanol films with bulky counterion, cause separation between crystals through 110 and 1-10 faces and therefore adsorbed water has an affinity to penetrate through CNC surface and show lower contact angle. Further with increasing amount of Methyl imidazole, contact angle is varying however, there is no special trend seen among these films. It is expected that methyl imidazole is adsorbing on 200 planes and there it is also weakening the hydrogen bonding between crystals and allowing water to penetrate through surfaces.

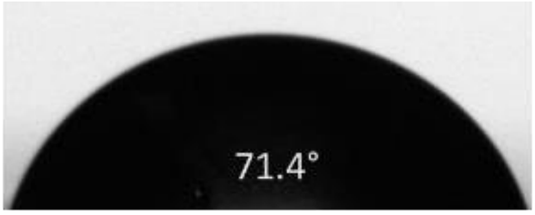

CNC-OSO_3H

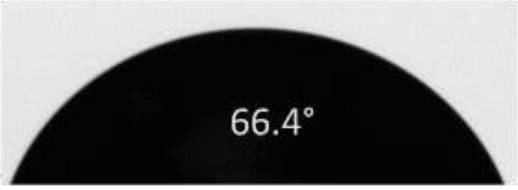

CNC-4 wt% TEOA

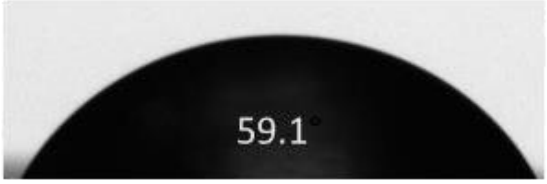

CNC-4 wt% TEOA 4.5 wt% MI (1:1:2)

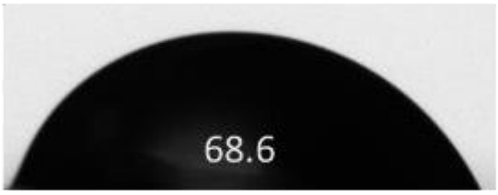

CNC-4 wt% TEOA 9 wt% MI (1:1:4)

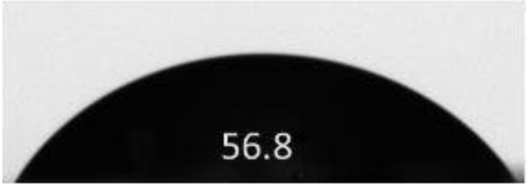

CNC-4 wt% TEOA 13.5 wt% MI (1:1:6)

Figure S7 - Contact angle measurements of CNC and modified CNC films.

S5. Thermal stability of CNC and modified CNC samples

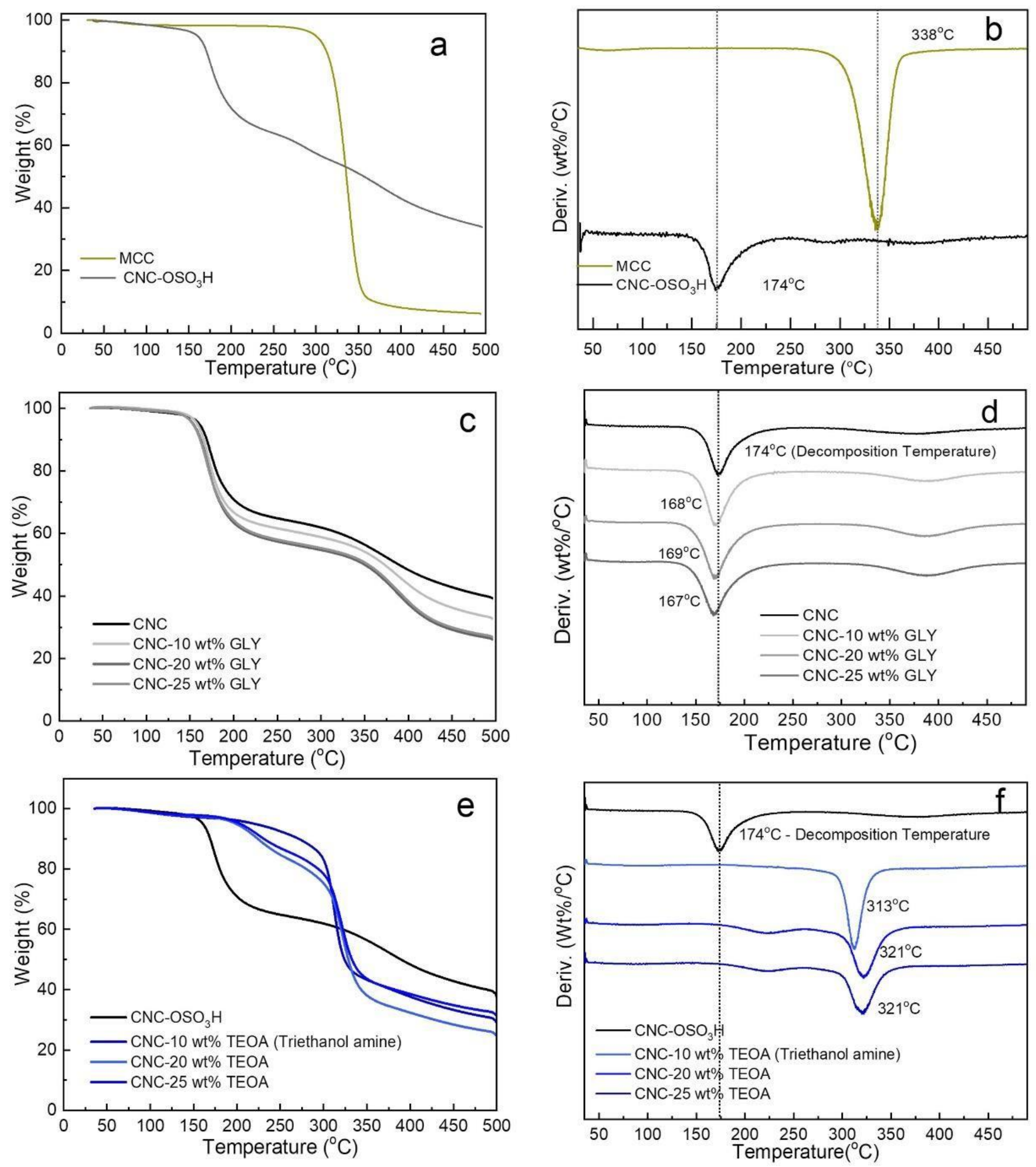

Figure S8 - (a) TGA and (**b)** DTGA of MCC and CNC samples **(c)** TGA and **(d)** DTGA of CNC and CNC-GLY samples **(e)** TGA and **(f)** DTGA of CNC and CNC-TEOA samples

S6. Mechanical properties of CNC-Glycerol (GLY) and CNC- Triethanol amine (TEOA) films

S.No.	CNC films	Tensile Strength (MPa)	Elongation at break (%)	Decomposition Temperature (°C)
**1**	CNC-OSO_3H	47 (3.2)*	1.3 (0.5)	174°
**2**	CNC- 10 wt%-Gly	37.4	2 (0.2)	168°
**3**	CNC- 20 wt% Gly	15	2 (0.4)	169°
**4**	CNC- 25 wt% Gly	18	2 (0.27)	167°
**5**	CNC-10 wt% TEOA	48.5 (3.9)	3.5 (0.2)	313°
**6**	CNC- 20 wt % TEOA	20.1 (2.4)	3.4 (0.26)	321°
**7**	CNC -25 wt % TEOA	15.7 (0.8)	2.6 (0.3)	321°

Table S3 - Mechanical and thermal properties of CNC and plasticized CNC films

Gly;glycerol and TEOA; triethanolamine
*Standard error of the mean values

S7. Synthesis of Cellulose nanocrystal from Microcrystalline cellulose

The cellulose nanocrystal suspensions were derived from Microcrystalline cellulose – (Avicell) in house using following schematic as described earlier[2, 5].

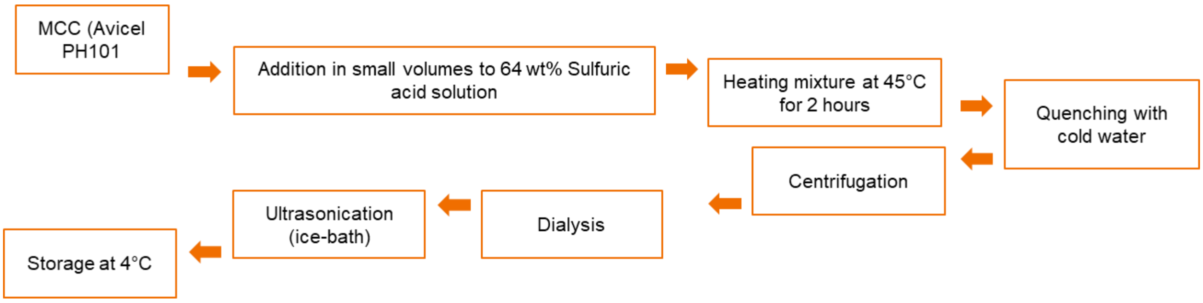

Figure S9 - Flow chart for preparing CNC from MCC

S8. Working Mechanism of TENG

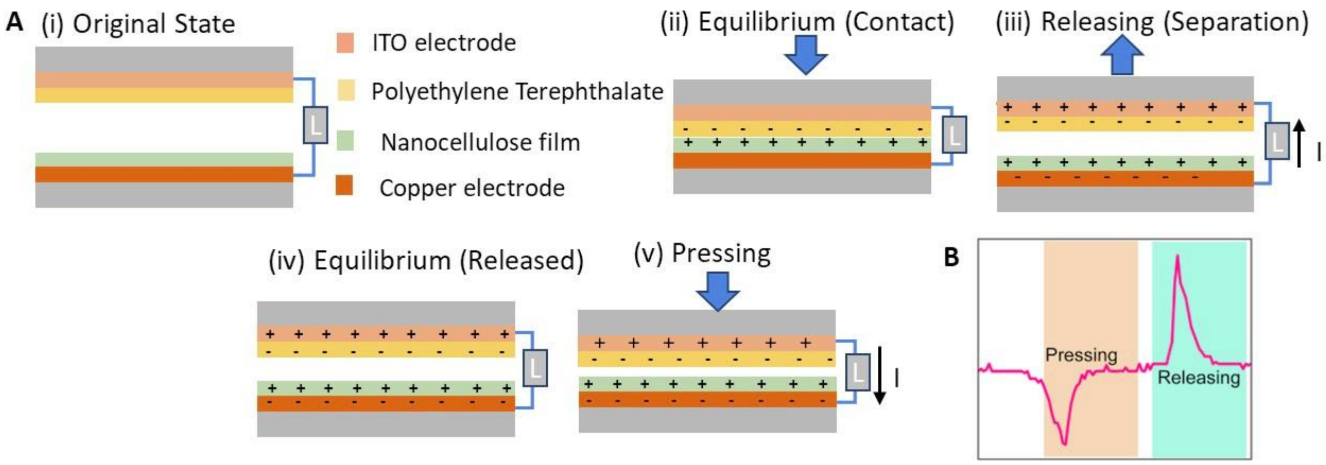

Figure S10 - Diagram of the (A) CNC-PET triboelectric nanogenerator composition and working mechanism (B) Standard TENG signal observed during pressing and releasing

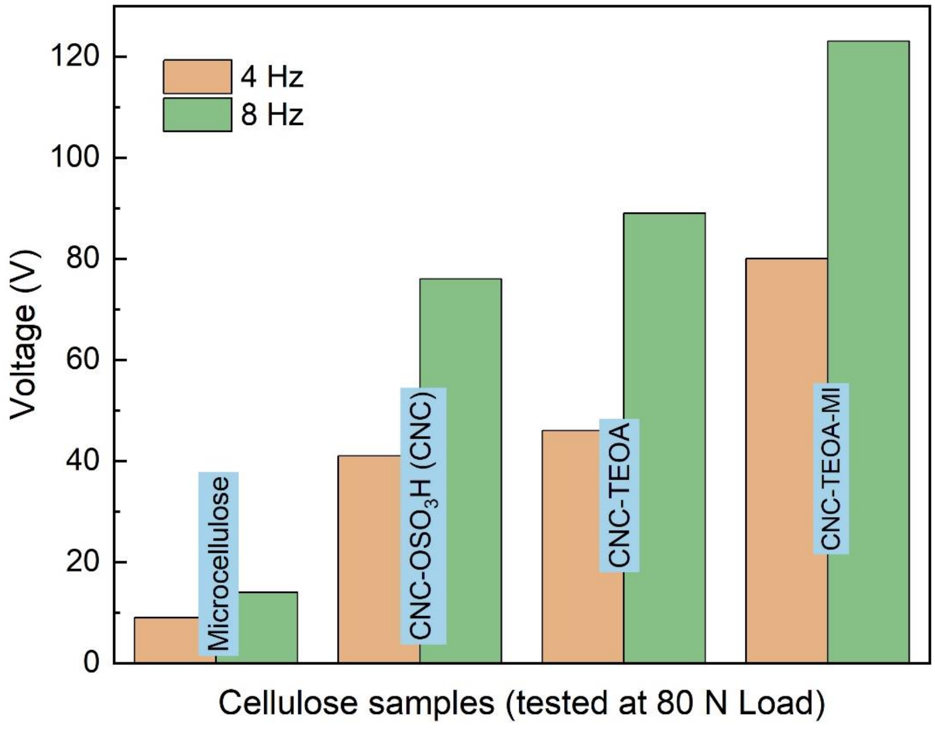

Figure S11 – Demonstration of clear increase in the output voltage with high frequency for different cellulose samples at given load of 80N

S9. Surface roughness of MCC, CNC and modified CNC films

3D optical images with surface roughness (μm) for different cellulose samples are provided below. The higher the Sq (root mean square height) in surface texture analysis, the greater the roughness. A higher Sq value indicates increased roughness, and vice versa.

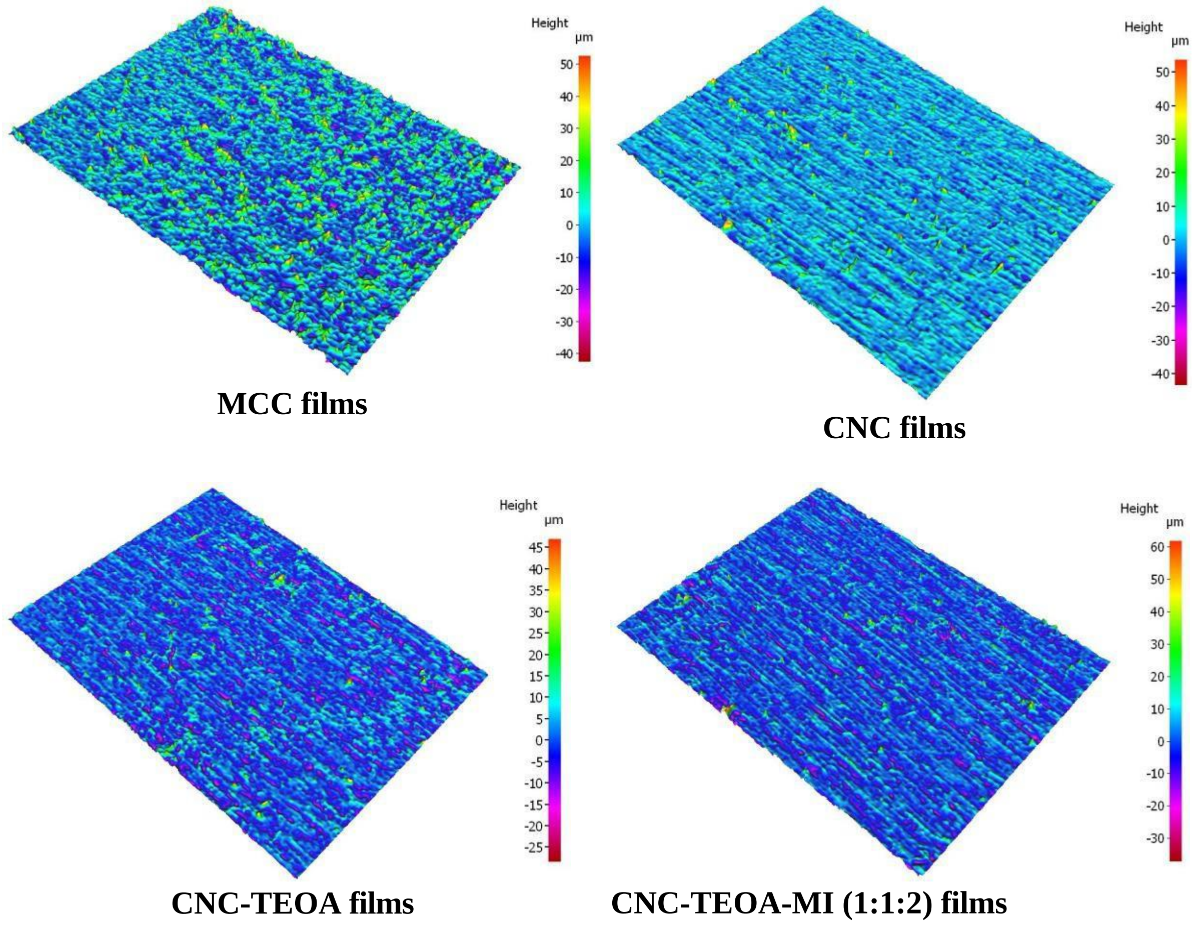

Figure S12 - 3D-optical images of different cellulose samples

Samples	Surface roughness (Sq) (μm)	Surface Charge	Dielectric constant
MCC	10.34	No Surface charge	3-4 [8]
CNC	5.6	-OSO_3H	4-6 [8]
CNC-TEOA	5.3	-OSO_3-Triethanolamine	Expected to be increased due to polarization
CNC-TEOA-MI	6	-SO_3-triethanolamine, adsorbed methyl imidazole	

Table S4 - Roughness data, surface charge and dielectric constant for MCC and different CNC samples

S10. Supporting video for figure 7B in main manuscript

The video shows the 'MMRG' logo (figure 7b, main manuscript) being illuminated by the power generated during the long-term stability test of the optimized PET-CNC TENG, conducted at a load of 40 N and a frequency of 4 Hz

References

(1) Daicho, K.; Saito, T.; Fujisawa, S.; Isogai, A. The Crystallinity of Nanocellulose: Dispersion-Induced Disordering of the Grain Boundary in Biologically Structured Cellulose. *ACS Applied Nano Materials* **2018**, *1* (10), 5774-5785. DOI: 10.1021/acsanm.8b01438

(2) Aggarwal, R.; Garg, A. K.; Saini, D.; Sonkar, S. K.; Sonker, A. K.; Westman, G. Cellulose Nanocrystals Derived from Microcrystalline Cellulose for Selective Removal of Janus Green Azo Dye. *Industrial & Engineering Chemistry Research* **2023**, *62* (1), 649-659. DOI: 10.1021/acs.iecr.2c03365

(3) Agarwal, U. P.; Ralph, S. A.; Baez, C.; Reiner, R. S.; Verrill, S. P. Effect of sample moisture content on XRD-estimated cellulose crystallinity index and crystallite size. *Cellulose* **2017**, *24* (5), 1971-1984. DOI: 10.1007/s10570-017-1259-0

(4) Schütz, C.; Bruckner, J. R.; Honorato-Rios, C.; Tosheva, Z.; Anyfantakis, M.; Lagerwall, J. P. F. From Equilibrium Liquid Crystal Formation and Kinetic Arrest to Photonic Bandgap Films Using Suspensions of Cellulose Nanocrystals. *Crystals* **2020**, *10* (3), 199.

(5) Tideland, H.; Feldhusen, J.; Sonker, A. K.; Westman, G. Bendable transparent films from cellulose nanocrystals–Study of surface and microstructure-property relationship. *Carbohydrate Polymer Technologies and Applications* **2023**, *6*, 100367. DOI: https://doi.org/10.1016/j.carpta.2023.100367

(6) Sørensen, B. E. A revised Michel-Lévy interference colour chart based on first-principles calculations. *European Journal of Mineralogy* **2012**, *25* (1), 5-10. DOI: 10.1127/0935-1221/2013/0025-2252 (acccessed 7/5/2022).

(7) Mendoza-Galván, A.; Tejeda-Galán, T.; Domínguez-Gómez, A. B.; Mauricio-Sánchez, R. A.; Järrendahl, K.; Arwin, H. Linear Birefringent Films of Cellulose Nanocrystals Produced by Dip-Coating. *Nanomaterials* **2019**, *9* (1), 45.

(8) Jones, S. H.; King, M. D.; Ward, A. D. Determining the unique refractive index properties of solid polystyrene aerosol using broadband Mie scattering from optically trapped beads. Phys. Chem. Chem. Phys. 2013, 15 (47), 20735-20741, 10.1039/C3CP53498G. DOI: 10.1039/C3CP53498G.

(9) Niskanen, I.; Suopajärvi, T.; Liimatainen, H.; Fabritius, T.; Heikkilä, R.; Thungström, G. Determining the complex refractive index of cellulose nanocrystals by combination of Beer-Lambert and immersion matching methods. Journal of Quantitative Spectroscopy and Radiative Transfer 2019, 235, 1-6. DOI: https://doi.org/10.1016/j.jqsrt.2019.06.023.

(10) Cranston, E. D.; Gray, D. G. Birefringence in spin-coated films containing cellulose nanocrystals. Colloids and Surfaces A: Physicochemical and Engineering Aspects 2008, 325 (1), 44-51. DOI: https://doi.org/10.1016/j.colsurfa.2008.04.042.

(11) Landry, V.; Alemdar, A.; Blanchet, P. Nanocrystalline Cellulose: Morphological, Physical, and Mechanical Properties. Forest Products Journal 2011, 61 (2), 104-112. DOI: 10.13073/0015-7473-61.2.104.

(12) Reid, M. S.; Villalobos, M.; Cranston, E. D. Cellulose nanocrystal interactions probed by thin film swelling to predict dispersibility. Nanoscale 2016, 8 (24), 12247-12257.
